# Characterization of Former Gestational Diabetes Mellitus: Prognostic, Therapeutic, and Predictive Aspects

**DOI:** 10.1155/2012/109038

**Published:** 2012-08-23

**Authors:** Andrea Tura, Alexandra Kautzky-Willer, Graziano Di Cianni, Yariv Yogev

**Affiliations:** ^1^Metabolic Unit, Institute of Biomedical Engineering, National Research Council, 35127 Padova, Italy; ^2^Gender Medicine Unit, Division of Endocrinology and Metabolism, Department of Internal Medicine III, Medical University of Vienna, 1090 Vienna, Austria; ^3^Department of Diabetes and Metabolic Diseases, Livorno Hospital, 57100 Livorno, Italy; ^4^Division of Obstetrics and Delivery Ward, Helen Schneider Hospital for Women, Rabin Medical Center, Sackler Faculty of Medicine, Tel Aviv University, 49100 Tel Aviv, Israel

## 1. Introduction

This special issue was focused on original research and review articles regarding the pathophysiology of glucose and lipid metabolism in women with previous gestational diabetes mellitus, as well as the identification of early markers for the risk of developing type 2 diabetes and related cardiovascular complications in that category of women. In addition, the study of the metabolic condition during pregnancy, that is, during overt gestational diabetes, was also of interest for the special issue. Some reports on educational programs for behavior modification and improved awareness of the disease were also included. In fact, it is known that the risk of developing type 2 diabetes is increased when the condition of previous gestational diabetes is accompanied by other factors, which predispose to diabetes also in different categories of subjects, like improper diet habits, sedentary lifestyle, obesity, or weight gain. The following paragraphs briefly present the articles included in this special issue. In the first section, we summarize the articles related to basic studies of pathophysiology, with special focus on the relationships between metabolic, endocrine, and cardiovascular parameters. The second section refers to articles more directly related to the disease care, including possible approaches for prevention and for improved clinical care and home support. In the last section some review articles are reported.

## 2. Pathophysiology/Metabolic, Endocrine, Cardiovascular Interactions

The study by S. Farhan et al. investigated the possible relationships between fetuin-A and gestational diabetes. Fetuin-A is calcification inhibitor, also interacting with insulin receptor tyrosine kinase, thus possibly causing insulin resistance. In fact, in human studies fetuin-A was found to be related to insulin resistance and metabolic syndrome, and linked to incident diabetes mellitus. In the women with gestational diabetes analyzed by S. Farhan et al., significant relationships between fetuin-A and parameters of insulin sensitivity or metabolic control were not observed, but a strong relationship was found with the early postpartum body mass index.

The study by E. Vitacolonna et al. investigated the prevalence of thyroid dysfunction and autoimmunity in women with gestational diabetes. They report that increased incidence of organ-specific autoimmunity towards endocrine cells other than beta-cells was described in type 1 diabetes, but recently some studies also reported increased incidence of thyroid autoimmunity in type 2 diabetes, thus suggesting that diabetes can trigger the onset of the thyroid autoimmune disorder. However, few studies evaluated the prevalence of thyroid dysfunction and autoimmunity in women with gestational diabetes. E. Vitacolonna et al. found that maternal hyperglycemia is a risk factor for later development of thyroid autoimmunity. In fact, during pregnancy they did not observe different prevalence in thyroid dysfunction and autoimmunity between gestational diabetic and normally pregnant women, but a significant increase in thyroid autoimmunity was seen after gestation in the women previously affected by gestational diabetes.

In the study by A. Sokup et al., the hypothesis that in women with a history of gestational diabetes, early endothelial dysfunction (2 to 24 months after delivery) is associated with the development of glucose dysregulation and other atherosclerosis risk factors was explored. A. Sokup et al. analyzed several parameters, with special focus to some parameters of endothelial dysfunction, such as the concentration of soluble E-selectin, vascular cell adhesion molecule-1 (VCAM-1), and intracellular adhesion molecule-1 (ICAM-1). They found that soluble VCAM-1 and some parameters of atherogenic dyslipidemia were associated with previous gestational diabetes independently of body mass index, insulin resistance, and fasting glucose, whereas soluble E-selectin correlated with metabolic syndrome components, but it was not independently associated with abnormal glucose regulation. They concluded that the atherogenic dyslipidemia may be a key metabolic predictor of cardiovascular risk in women with previous gestational diabetes.

The study by A. Ghio et al. aimed to assess if changes in insulin action and secretion during pregnancy are related to 1-hour plasma glucose concentration during an oral glucose tolerance test. According to the new international criteria of gestational diabetes (IADPSG criteria) each glucose value, that is, fasting, 1 hour, and 2 hours are equally but independently important and may present different pathophysiologic conditions. A. Ghio et al. found that for each 20 mg/dL increase in 1-hour plasma glucose concentration there was a concomitant impairment in insulin action and reduction in insulin secretion. In the authors' opinion, the alterations in insulin action and secretion are more apparent for elevations of the 1-hour plasma glucose than for other time samples of the oral glucose tolerance test. Thus, it is suggested that 1-hour value may provide a better parameter for risk stratification in pregnant women.

In the study by G. Pacini et al., the incretin effect was assessed in a group of women with a history of gestational diabetes who underwent both intravenous and oral glucose tolerance test immediately after partum. Since it is known that the incretin hormones contribute to insulin secretion after oral administration of glucose, the incretin effect was assessed by comparing in each individual the possible differences in beta-cell function between intravenous and oral glucose tolerance tests. G. Pacini et al. found that normotolerant women with previous gestational diabetes exhibit an incretin effect similar to that of healthy women, who had a normal pregnancy. At contrast, compromised incretin effect, proper of obese and type 2 diabetic subjects, characterizes women with previous gestational diabetes and current condition of impaired glucose tolerance. Prevention studies in women with a history of gestational diabetes using incretin-based therapies will clarify if these drugs may exert a specific benefit in this subgroup of subjects at increased risk of type 2 diabetes. 

## 3. Improved Prevention, Clinical and Home Care Approaches

The study by A. M. Ramos-Leví et al. proposed a model for the assessment of risk of developing gestational diabetes. A. M. Ramos-Leví et al. analyzed women attending prenatal care that were screened for gestational diabetes. Women completed a questionnaire on sociodemographic, anthropomorphic, and behavioral characteristics, and reproductive and medical history. The authors found that acting on modifiable factors, that is promoting healthy lifestyle habits such as moderate intake of coffee, low intake of biscuits, sugared drinks and red meats, and regular physical activity may represent a promising approach for the prevention of gestational diabetes. 

The article by E. Korpi-Hyövälti et al. describes a study aimed to the increase of postpartum testing, based on oral glucose tolerance tests and other associated factors, in women at risk for gestational diabetes. E. Korpi-Hyövälti et al. found that the most important explaining factor for compliance to the postpartum testing program was a special call or reminder from the central hospital. They concluded suggesting that communication between primary care providers, obstetrics and gynecology care providers, and endocrinologists should be encouraged, and a reminding system for primary care should be developed.

The report from A. Lapolla et al. presented some results from the DAWN (Diabetes Attitudes, Wishes and Needs) study. The DAWN study is a survey promoted by the International Diabetes Federation to recognize needs, perceptions, and feelings of people affected by diabetes mellitus, and identify possible areas of concern. Within the DAWN framework, A. Lapolla et al. performed a survey specifically addressed to women with gestational diabetes. The survey included questions on several topics, such as the general characteristics of the women, the evolution of gestational diabetes, the diet and lifestyle regime during gestation, the relationships with the gestational diabetes specialist and the other medical doctors, the quality of family support, and the feelings related to the diagnosis of gestational diabetes. It was found that the large majority of women were satisfied with the quality of care, though the degree of cooperation between diabetes specialists and gynecologists was considered sometimes unsatisfactory. In addition, some women reported troubles in following the prescribed program of glucose self-monitoring and the suggested dietary regimen.

The study by H. D. McIntyre et al. proposed a home-based exercise program with telephone support for the early postpartum period in women with recent gestational diabetes. H. D. McIntyre et al. found that the physical activity (walking being the predominant type) was increased in the women included in the educational program compared to women receiving usual care support. However, it was acknowledged that significant changes were not observed in body weight and composition, or in the main parameters of glucose metabolism, probably due to the relatively short duration of the study.

The study by B. Valentini et al. suggests that when a diet for immigrant women with gestational diabetes is prescribed, their different traditional eating habits should be considered. To this purpose, B. Valentini et al. proposed a food plan including dishes typical of the foreign women's original countries (the “ethnic meal plan”). They compared the ethnic meal plan to a standard meal plan in immigrant women with gestational diabetes. They found that the ethnic approach to diet has positive effect on the outcome of pregnancy, as the ethnic meal plan group had babies with a lower birth weight, and no cases of macrosomia were observed. They concluded that the ethnic method could be considered a valid approach to the nutritional management of immigrant pregnant women with gestational diabetes.

The study by X. Jian Yun et al. assessed to what extent short-term use of corticosteroids for fetal lung maturation affects fasting blood glucose and insulin levels in normal singleton pregnancies, normal twin pregnancies, and pregnancies with impaired glucose tolerance condition but not requiring insulin treatment. Specifically, X. Jian Yun et al. investigated whether glucose and insulin levels differ after the administration of dexamethasone. They found that the degree of modification of the maternal fasting plasma glucose and insulin levels determined by dexamethasone was correlated with the basic maternal glucose metabolic condition, and may be correlated with twin pregnancy status. They concluded that blood glucose levels of twin pregnancies, and those with impaired glucose tolerance, should be closely monitored during the use of dexamethasone. 

## 4. Review Articles

The article by G. E. Rice et al. is a review on the benefits of screening for type 2 diabetes in women with a previous history of gestational diabetes. In fact, it is known that women with previous gestational diabetes are at a greater risk of developing type 2 diabetes within 10 to 20 years of their index pregnancy. However, there is still the need for better early detection of predisposition to the development of the disease. G. E. Rice et al. discuss some screening approaches, with special attention to preconception screening strategies. Furthermore, as regards preconception programs in women already with overt diabetes, the authors report that several studies have documented evidence of positive financial returns for preconception counseling, mainly based on savings in hospitalisation costs.

One article by N. Vrachnis et al. is a review on the role of adipokines and other inflammatory mediators in gestational diabetes. Previous gestational diabetes has been associated with future development of both type 2 diabetes and metabolic syndrome. N. Vrachnis et al. report that the pathogenesis and risk factors implicated in the later development of these conditions are not as yet fully understood, but scientific research has recently focused on a group of substances produced mainly by adipose tissue called adipokines (such as adiponectin, leptin, retinol-binding protein-4 (RBP-4), and resistin). These substances as well as other inflammatory mediators (CRP, IL-6, PAI-1, and TNF-**α**) seem to play an important role in glucose tolerance and insulin sensitivity dysregulation in women with previous gestational diabetes. In fact, such women are characterized by chronic subclinical inflammation, which is associated with insulin resistance and abnormality in glucose metabolism.

Another review article by N. Vrachnis et al. revised the role of possible markers of risk for cardiovascular diseases in women with a history of previous gestational diabetes. In fact, it is known that previous gestational diabetes can increase the risk of developing not only type 2 diabetes, but also cardiovascular diseases independently of a diagnosis of diabetes. N. Vrachnis et al. report and discuss several markers for cardiovascular diseases risk, such as insulin resistance, hyperlipidemia, increased levels of ICAM-1, VCAM-1, E-selectin, and low levels of adiponectin.

## 5. Conclusions

Several topics have been addressed in the studies included in this special issue dedicated to gestational diabetes. The focus of these studies was not limited to the assessment of the main parameters in glucose metabolism, such as insulin sensitivity, insulin secretion, and beta-cell function, but also to the analysis of the interactions between such metabolic variables and different hormones and parameters, such as fetuin-A, thyroid parameters, and cardiovascular markers. In addition, several studies were specifically focused on different aspects of care, including strategies to enhance adherence to prescriptions in terms of glucose testing, appropriate diet, and improved general lifestyle, for prevention of both type 2 diabetes and cardiovascular diseases. The variety of the presented studies shows that gestational diabetes is a metabolic condition of primary clinical interest. In our opinion, some other research topics not specifically addressed in this special issue, which will gain increasing relevance, are the study of offspring of women with a history of gestational diabetes, fetal programming research for improved understanding of intrauterine environment influences on both short and long-term fetal outcomes, and epigenetic studies to identify the processes that drive the evolution of an individual phenotype from the genome, and investigate to what extent the intrauterine environment determines how these genes act. In summary, we believe that in the next future several new interesting studies will come in the field of gestational diabetes.

*Andrea Tura* *Andrea Tura* *Alexandra Kautzky-Willer* *Alexandra Kautzky-Willer* *Graziano Di Cianni* *Graziano Di Cianni* *Yariv Yogev* *Yariv Yogev*

